# Review of Sensor-Based Subgrade Distress Identifications

**DOI:** 10.3390/s24092825

**Published:** 2024-04-29

**Authors:** Zhiheng Cheng, Zhengjian Xie, Mingzhao Wei, Yuqing Peng, Cong Du, Yuan Tian, Xiuguang Song

**Affiliations:** 1 School of Qilu Transportation, Shandong University, Jinan 250061, China; 202235453@mail.sdu.edu.cn (Z.C.); 202335514@mail.sdu.edu.cn (M.W.); songxiuguang@sdu.edu.cn (X.S.); 2 CCCC-FHDI Engineering Co., Ltd., Guangzhou 510220, China; xiezj@fhdigz.com; 3 School of Traffic and Transportation, Lanzhou Jiaotong University, Lanzhou 730070, China; 11220014@stu.lzjtu.edu.cn

**Keywords:** subgrade distress, detection sensor, data processing

## Abstract

The attributes of diversity and concealment pose formidable challenges in the accurate detection and efficacious management of distresses within subgrade structures. The onset of subgrade distresses may precipitate structural degradation, thereby amplifying the frequency of traffic incidents and instigating economic ramifications. Accurate and timely detection of subgrade distresses is essential for maintaining and repairing road sections with existing distresses. This helps to prolong the service life of road infrastructure and reduce financial burden. In recent years, the advent of numerous novel technologies and methodologies has propelled significant advancements in subgrade distress detection. Therefore, this review delineates a concentrated examination of subgrade distress detection, methodically consolidating and presenting various techniques while dissecting their respective merits and constraints. By furnishing comprehensive guidance on subgrade distress detection, this review facilitates the expedient identification and targeted treatment of subgrade distresses, thereby fortifying safety and enhancing durability. The pivotal role of this review in bolstering the construction and operational facets of transportation infrastructure is underscored.

## 1. Introduction

The subgrade, constituting a fundamental component of road infrastructure, spans the entirety of road construction endeavors [1,2]. Primarily, it functions as the foundation of the pavement, bearing the load imposed by vehicular traffic. Simultaneously, it plays a crucial role in mitigating road settlement and deformation. Recognized as the cornerstone of highway engineering, the subgrade shoulders the responsibility of sustaining the road system’s weight and furnishing steadfast foundational support, thereby safeguarding the highway’s safety, comfort, and reliability. Attaining the optimal design and upkeep of the subgrade is imperative as it ensures the stability, durability, and load-bearing capacity of the road, consequently fostering a conducive, expeditious, and safe transportation milieu for travels [3,4].

Detecting road distresses holds the potential to improve the service performance of the subgrade, prolong the service life of the road, and curtail associated costs. Over time, as roads undergo extended periods of use, a plethora of defects may manifest within the subgrade, encompassing embankment and subgrade irregularities [5], deficiencies in drainage facilities [6], slope defects [7], pre-existing flaws in protective and retaining structures [8], as well as shoulder defects [9]. Failure to promptly identify and rectify these imperfections may engender risks to traffic safety, escalate operational expenses, and directly impact the road’s longevity. Therefore, scientific, efficient, and precise methodologies for detecting subgrade defects are imperative. Such approaches facilitate the identification, documentation, and analysis of surface irregularities, furnishing a foundational framework for informed decision-making pertaining to road maintenance and repair endeavors [10].

**Figure 1:**
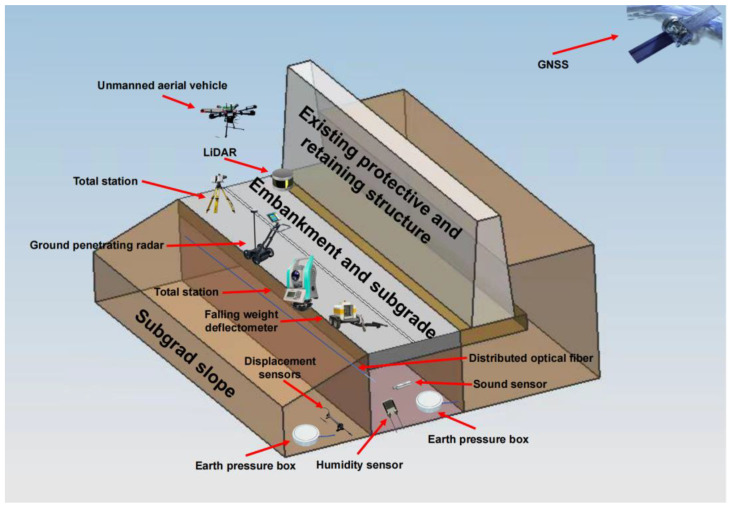
Schematic diagram of the roadbed structure and the corresponding sensor layout.

Presently, the detection of subgrade defects is beset by challenges such as low efficiency in manual inspection, subjectivity and inconsistency, reliance on specialized technical personnel, and inadequate periodic surveillance. To surmount these limitations, advanced technologies and methodologies, including ground-penetrating radar, level instruments, falling weight deflectometers, and diverse embedded sensors, are employed to realize automated and efficient subgrade defect detection and evaluation, thereby bolstering precision and efficiency [11]. This review researches various methodologies for subgrade defect detection, data processing, and modeling analysis. Notably, owing to minimal traffic impact and difficulty associated with large-scale detection of shoulder areas, research on shoulder defects remains relatively scant, with a predominant reliance on manual inspection [12]. In alignment with the composition of the subgrade, this paper classifies subgrade defects into categories such as embankment and subgrade distresses, drainage facility distresses, subgrade slope distresses, and deficiencies in existing protective and retaining structures. The subgrade structure and corresponding sensor layout are shown in Figure 1.

Comprehensive investigations and analyses of detection methodologies and their respective strengths and limitations are conducted for these distinct defect types. This provides comprehensive guidance for subgrade defect detection, thereby facilitating timely detection and targeted remediation, and augmenting road safety and longevity. This endeavor assumes a pivotal role in improving the overall standard of construction and operational practices within transportation infrastructure, while also laying the foundation for future advancements in the high-precision detection of subgrade defects.

## 2. Embankment and Subgrade

### 2.1. Distress

Subgrade-based deformation refers to a range of deformation phenomena experienced by road-based structures during their use. These phenomena primarily include subgrade settlement [13], subgrade frost heaving [14], weak subgrade, and other related issues [15]. The occurrence of these deformation ailments not only diminishes driving comfort and affects the overall aesthetics of the road but also poses risks to driving-related safety and reduces the service life of the road. The instruments for the disease-related detection of the embankment and subgrade and the reference article numbers are shown in Table 1.

Subgrade settlement is caused by many factors, such as uneven soil layer, load, moisture, natural factors, construction factors, and design defects [16]. Nowadays, common subgrade settlement detection instruments include ground-penetrating radar, total station, interferometric synthetic aperture radar, distributed optical fiber, and other equipment and software. These instruments can judge whether the subgrade has settled by measuring the road surface elevation, pressure, vibration, and deformation [17].

The frost heaving of the subgrade refers to a geological phenomenon where the moisture in the subgrade soil layer will affect the subgrade due to the volume expansion of ice when it encounters low temperatures. The change in groundwater depth is one of the important factors leading to subgrade frost heaving [18,19]. At present, the detection methods of subgrade frost heave distresses include ground-penetrating radar, falling weight deflectometers, and other equipment, or sensors such as temperature sensors, moisture sensors, and frost heave meters embedded in the subgrade.

Weak subgrade distress refers to a common distress of subgrade deformation, settlement, or instability under load [20]. This type of distress usually occurs in soft foundation soil or subgrade deformation caused by improper construction. There are many reasons for weak subgrade distresses, including loose soil, high water content, insufficient compaction, and poor construction quality [21]. At present, the main instrument for detecting subgrade strength is the falling weight deflectometer.

**Table 1:** Representative equipment for embankment and subgrade.

Structure Type	Distress	Sensor	References
Embankment and subgrade	Subgrade settlement	Ground-penetrating radar (GPR)	[22,23,24,25,26]
		Total station	[27,28,29,30,31]
		InSAR	[32,33,34,35,36]
		Distributed optical fiber	[37,38,39,40,41]
	Subgrade frost heaving	Ground-penetrating radar (GPR)	[42,43,44,45]
		Multi-sensor embedding	[46,47,48,49,50]
	Weak subgrade	Falling weight deflectometer (FWD)	[51,52,53,54,55,56]

### 2.2. Monitoring Technology

Table 1 summarizes the representative research findings related to embankment and subgrade distress detection, and the detailed applications are elaborated in the following subsections.

#### 2.2.1. Ground-Penetrating Radar

**Figure 2:**
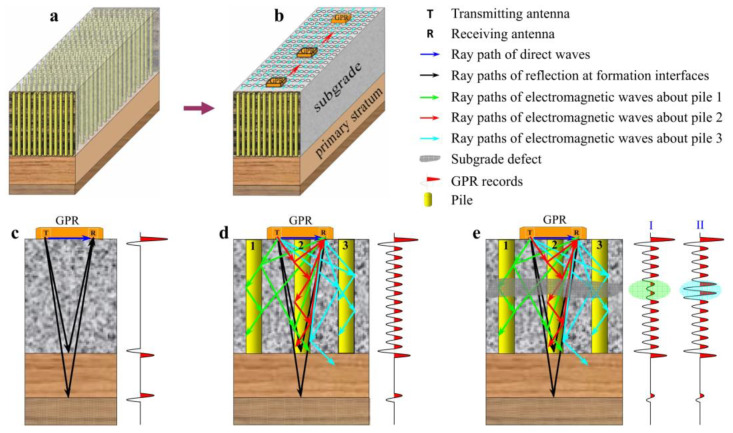
Schematic diagram of ground-penetrating radar detection. (**a**) Geological model of subgrade reinforced by pile groups. (**b**) Equivalent geological model of subgrade reinforced by pile groups. (**c**) Stratum without pile, (**d**) stratum with complete piles, (**e**) stratum with defective piles and theirs GPR response characteristics. (figures are reproduced from Shen et al. [26]).

Ground-penetrating radar (GPR) is a non-invasive technology used for underground detection and imaging, employing electromagnetic waves. The underlying principle involves transmitting electromagnetic waves into the ground. As these waves encounter changes in the underground structure caused by subgrade settlement or subgrade frost heaving, such as variations in soil density and the presence of sediments, they undergo reflection or refraction. The received and analyzed signals from the ground-penetrating radar provide information on the intensity and time, enabling the determination of the position, shape, and properties of the detected underground objects or structures. For subgrade settlement distresses, through data processing and interpretation, it becomes possible to assess the settlement status of the subgrade accurately and evaluate the degree of settlement [57]. To process ground-penetrating radar data, Liu et al. [22] proposed a novel deep-learning method for processing GPR data, namely the CRNN network. This network model combines the Convolutional Neural Network (CNN) and Recurrent Neural Network (RNN). In this model, the CNN processes the raw GPR waveform data from signal channels, while the RNN processes features from multiple channels. The experimental results demonstrate that the developed deep learning method improves the efficiency and accuracy of railway subgrade condition assessment. Similarly, Elsecy Ahmed and colleagues [23] have utilized ground-penetrating radar to detect flexible and rigid pavements. Sarlah Nikolaj et al. [24] have combined robots with ground-penetrating radar to detect settlements on urban roads, demonstrating their robustness. With regards to distress detection in the subgrade, Yuqiqin et al. [25] have proposed an algorithm based on continuous wavelet transform. Moreover, Shen Hongyan and others [26] have developed attribute analysis technology for ground-penetrating radar and established a technical system for evaluating the quality of ground treatments. The working diagram of ground penetrating radar is shown in Figure 2.

According to the actual situation of defect detection and foundation treatment quality evaluation of mixing piles and the characteristics of collected ground-penetrating radar data, in addition to zero correction, gain recovery, inter-channel, and in-channel energy compensation, interference noise suppression and multiple wave attenuation should be emphasized. Its purpose is to ensure high-quality GPR data processing results without losing effective information. In addition, the ground-penetrating radar data obtained under the condition of an undulating surface needs terrain correction. Thus, it has been further proven that the results of ground-penetrating radar combined with single-channel waveform, multi-channel profile, and attributes can effectively detect the defects and stratum structure after foundation treatment. The research results of this paper provide a fast, efficient, and economical technical solution for software quality evaluation.

For subgrade frost heaving, Sabbar Abdalsh Salih [27] and others successfully demonstrated the feasibility of detecting low water levels in shallow water by using ground-penetrating radar. Han Hongwei [28] and others successfully used ground-penetrating radar to detect moisture and the existence of ice crystals under the ground depending on the different transmission speeds of electromagnetic waves in different media. Xu Yi [29] and others used ground-penetrating radar to accurately predict the leakage position at the joint of the underground diaphragm wall, used MATLAB to calculate the average wave velocity amplitude and single channel signal of electromagnetic wave velocity of ground-penetrating radar at different mileage, and drew the trend diagram of average wave velocity amplitude with mileage and the corresponding curve of the relationship between electromagnetic wave amplitude and radar depth. Song Wenlong [30] and others put forward a pixel-scale method to measure subgrade humidity by using ground-penetrating radar. Under 95% confidence, the four survey lines of ground-penetrating radar met the requirements, and the relative error of each plot was 5%. Liu Sixin [31] and others used the common midpoint measurement of ground-penetrating radar to estimate the propagation speed of electromagnetic waves in subgrade applications, established a synthetic model to simulate the railway subgrade structure, and formed a long moisture content curve of the railway subgrade. The synthetic ordinary midpoint aggregates obtained from shallow and thin layers are seriously disturbed by multiple waves and refracted waves, which makes the conventional velocity analysis unable to provide accurate velocity. Through the analysis of the numerical simulation results, it was found that the main reflected wave, multi-wave, and refracted wave are dominant in different migration ranges of the common midpoint set. Therefore, an optimal aggregation solution in a certain offset range dominated by the main reflected wave is used to calculate the velocity spectrum and extract the accurate velocity of the subgrade model. The relative dielectric constant of the corresponding layer is calculated after converting the stacking speed into the spacing speed. Then, the moisture content is obtained by the formula, which represents the relationship between the dielectric constant and moisture content. Finally, we apply the best collection scheme and the above explanation process to the ground-penetrating radar data collected on the railway site and form the long moisture content distribution map of the railway subgrade.

#### 2.2.2. Total Station

Total station is a kind of high-precision measuring equipment, which utilizes an internal laser transmitter and receiver to scan the measuring range and measure the angle and distance data of the point to be measured by rotating the horizontal and vertical disks. It can measure the measuring points at different positions on the subgrade in all directions and provide high-precision position and angle data. By conducting numerous observations and data analysis, the settlement of the subgrade can be evaluated.

Jiang Jiqing [32] proposed a real-time recognition method based on a Gated Recurrent Unit (GRU) neural network for monitoring differential settlement in railway foundations. This method utilizes a GRU network to establish the nonlinear relationship between the vertical acceleration of the vehicle and the differential settlement of the railway foundation. The experimental results demonstrate that the GRU neural network can accurately identify the longitudinal continuous differential settlement distribution curve of the railway foundation through real-time vibration responses of the vehicle and the railway. Caolin Qing et al. [33] constructed a single-mode settlement curve of subgrade settlement based on the total station for the construction section of the frozen soil layer. Wang Xin et al. [34] analyzed the settlement and deformation laws of the reinforced area and the lower layer of the pile-supported reinforced embankment, as well as the settlement laws of the transverse and longitudinal sections of the subgrade according to the subgrade data obtained by the total station. Zhou Shunhua et al. [35], given subgrade settlement distresses in soft soil areas with high compressibility and low permeability, used the total station to collect road section data and introduced the settlement evolution and settlement control effects of different treatment methods. Zheng Gang et al. [36] used a total station (DLZ6-1, with eight monitoring points) to monitor soil settlement at different distances. When the total station is used to detect subgrade settlement, the appropriate measuring points are selected first, and the total station is placed on one of them.

**Figure 3:**
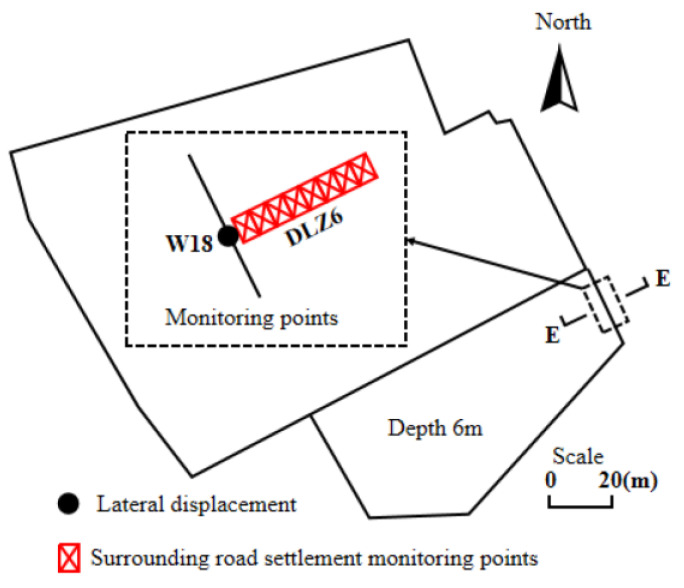
The project site and instrumentation layout (figures are reproduced from Zheng et al. [36]).

The total station Is calibrated by leveling feet and level meters to ensure its levelness. Then, the laser or electronic ranging function of the total station is used to accurately locate the measuring target and record the relevant data. After that, the total station is moved to the next measuring point, and the above steps are repeated. Finally, the settlement of the subgrade is calculated by processing and analyzing the collected data. As a traditional measuring instrument in the engineering field, the total station is constantly improving its accuracy and automation, making it easier to operate. Modern total stations have achieved digital, automatic, intelligent, and other technological innovations, which have improved measurement efficiency and accuracy. Therefore, the application prospect of total station in detecting subgrade settlement is broad and has been widely popularized. The site layout and installation diagram of the total station is shown in Figure 3.

#### 2.2.3. Interferometric Synthetic Aperture Radar

InSAR technology, also known as Interferometric Synthetic Aperture Radar, is a method used to monitor ground deformation using satellite radar. By comparing and analyzing two or more satellite radar images, it enables accurate monitoring of ground deformation. In terms of detecting subgrade settlement, InSAR technology can determine changes in subgrade surface elevation by comparing two satellite radar images, thereby identifying the occurrence of subgrade settlement. Compared to the GNSS (Global Navigation Satellite System), InSAR technology has the advantages of high precision, wide coverage, and non-contact measurement, providing more accurate and comprehensive monitoring data for subgrade settlement.

**Figure 4:**
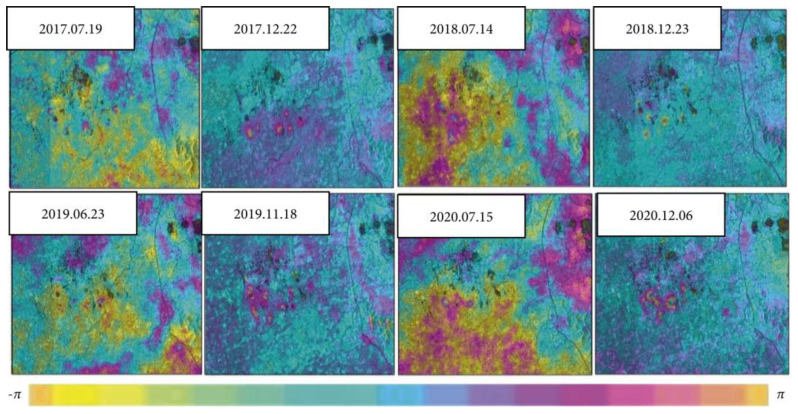
Interferogram of goaf in the Expressway (figures are reproduced from Tao et al. [41]).

Liu Hui et al. [37] utilized multi-time InSAR technology to monitor the deformation of the entire section of the Qinghai-Tibet Railway, detecting uneven settlement of the subgrade in space and seasonal variation in the subgrade over time. Liu Hui et al. [38] used InSAR technology to analyze the longitudinal and cross sections of four key monitoring sections and discovered uneven overall settlement patterns. Gagliardi Valerio et al. [39] mentioned that InSAR technology is one of the ground non-destructive testing methods and has been successfully applied for decades, achieving a high standard in terms of data quality and accuracy. Shi Min et al. [40] pointed out that InSAR technology has been successfully used to study the long-term micro-deformation of linear characteristics under all terrain conditions and large spatial scales. Tao Wenbin et al. [41] investigated the stability of an expressway goaf using InSAR technology and field investigation. Initially, the stability of the goaf around the route was predicted through field investigation. Then, satellite images were processed to extract subsidence data of mined-out areas from 2017 to 2021. Finally, the stability of risk points was analyzed. Figure 4 shows the road elevation data detected by the InSAR device over time.

According to the four-year settlement data extracted by InSar technology, two settlement risk areas were found, and the settlement areas showed an obvious inverted “convex” shape. The residual settlement in the next 15 years is predicted by the data collected by InSAR technology. Engineering practice shows that InSAR technology can effectively analyze the surface deformation of mined-out areas, which is of great practical significance for optimizing the selection of special geological routes of expressways.

#### 2.2.4. Distributed Optical Fiber

The principle of distributed optical fiber detection of subgrade settlement is to monitor subgrade deformation in real-time by using optical fiber sensing technology. An optical fiber is buried in the subgrade, and a continuous laser signal is sent into the optical fiber through a laser light source. When the optical signal changes physically with the medium around the optical fiber, such as stretching or compression caused by subgrade settlement, the scattering loss of the optical signal will change. By detecting the intensity and time delay of the scattered light signal, the deformation at various positions along the optical fiber can be determined to realize the continuous monitoring of the whole subgrade.

**Figure 5:**
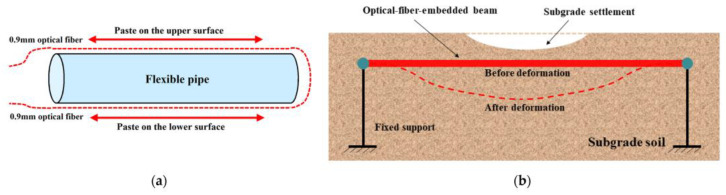
Schematic diagram of distributed fiber inspection. (**a**) Setup and (**b**) working principle of the OFEB (figures are reproduced from Li et al. [44]).

Sun Fan [42] and others put forward a new foundation treatment technology supported by optical fiber sensing technology and made a comprehensive evaluation. With the support of optical fiber sensing technology, the problems of foundation construction can be solved, and the phenomena of pavement settlement and cracking can be avoided to ensure the overall safety of the road. Weng Xiaolin et al. [43] used distributed optical fibers to measure the strain characteristics in the top and bottom layers of cement concrete pavement, which were affected by different foundation settlement degrees. Li Zhen [44,45] and others developed a distributed optical fiber and proposed a method to measure and calculate the beam deformation. A calibration test and a subgrade similarity model test were carried out to study the applicability and monitoring accuracy of the distributed optical fiber. The usage and operation diagram of distributed optical fiber is shown in Figure 5.

Through data analysis, it was concluded that distributed optical fiber can accurately measure beam deflection, and the maximum relative error between optical fiber and displacement sensor is about 5%. Distributed optical fiber is directly embedded in the similarity model of the subgrade to monitor the settlement. It was found that the deflection deformation of distributed optical fiber is close to subgrade settlement in a certain settlement range, and the relative error is less than 8.1%. Therefore, distributed optical fibers can be used to measure large-scale distributed settlement in subgrade. Numerical simulation is carried out to determine the appropriate beam size and material design parameters, thus expanding the measurement range before the distributed optical fiber is decoupled from the soil. Based on the preliminary experiments in this study, the measurement range and accuracy of distributed optical fiber are expanded so that settlement monitoring can be studied further.

#### 2.2.5. Multi-Sensor Embedding

When monitoring subgrade frost heaving distresses, various sensors such as temperature sensors and humidity sensors can be used for monitoring. The temperature sensor can measure the change in the temperature of the pavement and subgrade in real time, which provides the basic data for the formation of frost-heaving distresses. The humidity sensor can monitor the moisture content in the subgrade and grasp the humidity of the subgrade in time. The data obtained by these sensors can be analyzed and compared to judge whether there is frost heaving risk in the subgrade and take preventive measures in time. At the same time, combined with other sensors and data processing technology, it can also monitor the changes in subgrade settlement and displacement, comprehensively evaluate the health status of the subgrade, and provide effective support for the maintenance and management of subgrade engineering.

**Figure 6:**
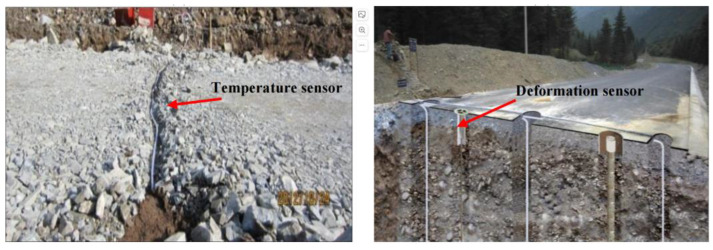
Sensor embedment diagram (figures are reproduced from Wu et al. [50]).

Xu Zhibo et al. [46] set up temperature sensors, humidity sensors, and settlement observation points on the permafrost subgrade and regularly used temperature instruments and humidity reading instruments to collect the temperature and humidity of the subgrade part, respectively. Huang Xuebang et al. [47] used temperature sensors and displacement sensors in the model to carry out freeze–thaw experiments to study the influence of repeated freeze–thaw on the performance of subgrade structures. Oyeyi Abimbola et al. [48] installed temperature and humidity sensors in road sections, which were placed in the middle of each pavement layer to measure the temperature and humidity of the layer, respectively. Huang Shaoping [49] simulated the structure of permeable subgrade in a constant-temperature container, installed the temperature sensor, conductance sensor, and moisture sensor in the constant-temperature container, connected them to a data recorder for data recording, and then reduced the temperature of the constant-temperature container to collect data on the changes in subgrade frost heaving. Wu Libo et al. [50] studied frost heaving deformation during a two-year monitoring period through field tests based on temperature sensors and deformation sensors. The embedment methods of various sensors are shown in Figure 6.

By analyzing the data collected by sensors in two years and applying the frost heaving model, the growth of ice crystals is explored, and the quantitative prediction of frost heaving is realized according to the experimental measurement results.

#### 2.2.6. Falling Weight Deflectometer

The falling weight deflectometer is a tool used to detect pavement distress by impacting a falling weight and measuring the deformation of the pavement. It can accurately and promptly identify issues such as pavement settlement and cracks, providing valuable insights for pavement maintenance and repair, thus ensuring the safety and stability of the subgrade.

Xu Changchun [51] and others utilized a falling weight deflectometer to test the elasticity of highway subgrade and proposed a comprehensive evaluation method for the compaction uniformity of highway subgrade based on the backpropagation (BP) neural network. Experimental results have demonstrated that this method can be effectively used for the accurate detection of weak subgrade distresses. Yao Yongsheng et al. [52] put forward the calculation and control method of the equivalent elastic modulus of subgrade top surface under non-uniform stress distribution and carried out the field verification test with a drop weight deflectometer. The results show that the calculation method of the equivalent elastic modulus of subgrade proposed in this study is reasonable and effective. Csaba Tóth et al. [53] proposed a calculation method based on the drop weight deflectometer, which can calculate the strength of each layer according to the deflection data of this non-destructive data acquisition method. Heejun Lee et al. [54] used a falling weight deflectometer to evaluate the strength of subgrade layers and the potential distresses. Sylwester M. Grajewski [55] determined the predicted primary deformation modulus according to the measurement results of a lightweight drop weight of 10 kg. After collecting deflection data, regression analysis was performed on 245 measured data points, which were carried out on 46 different road sections. Different regression models were tested, including linear models, logarithmic models, polynomial models, exponential models, and power models. The results show that the bearing capacity and compactness of the road subgrade can be inferred from the dynamic deformation modulus, and the weak subgrade distresses can be further detected. Li Danfeng [56] and others put forward an improved measurement method of subgrade bearing capacity based on dynamic modulus control and a portable drop weight deflectometer. Through theoretical analysis, the detection range of the falling weight deflectometer is determined and the data of the site are collected. Based on the collected data, the finite element numerical model of the subgrade dynamic stress field is established, and the spatial distribution and attenuation law of the subgrade dynamic stress field in the depth direction and horizontal direction are determined. The root cause of the difference between the static index and the dynamic index is discussed. By using the improved method, a prediction model with dynamic bending as the detection index and static resilience modulus as the control index is established. The model improves the detection accuracy and efficiency of weak subgrade and verifies the effectiveness of the improved method.

## 3. Drainage Facilities

### 3.1. Distress

Subgrade drainage refers to the measures taken in the process of road construction to guide the planning of rainfall or accumulated water out of the road area through the drainage system [58]. Subgrade drainage distresses mainly include poor runoff, subgrade pipeline leakage, erosion, collapse, and other situations, which will have a greater impact on road traffic. The instruments for disease-related detection of the drainage facilities and the reference article numbers are shown in Table 2.

At present, a considerable number of pipelines pass through the subgrade, and pipeline leakage has a great negative impact on the subgrade and even the road structure. The causes of pipeline leakage mainly include pipeline aging, corrosion, construction or design defects, third-party damage, and natural disasters. In long-term operation, pipeline materials will experience aging and corrosion, resulting in the thinning of the pipe wall, cracks, or loopholes, which will lead to leakage. At present, the equipment for monitoring pipeline leakage distresses includes sound sensors, humidity sensors, and ground-penetrating radar.

### 3.2. Monitoring Technology

Table 2 summarizes the representative research findings related to drainage facility distress detection, and the detailed applications are elaborated in the following subsections.

#### 3.2.1. Sound Sensor

Use special equipment to monitor the sound changes around the pipeline. When the pipeline leaks, it will produce specific sound signals, which can be analyzed to determine whether there is leakage.

Zhi Baoyu [59] and others developed an AlN-based MEMS hydrophone with miniaturization, low cost, low power consumption, and high sensitivity to monitor the leakage of water pipelines. Zaqretdinov Ayrat [60] and others analyzed the acoustic signals recorded from the pipeline surface under different leaks and water pressures and established a relationship between the size of the leakage and the Hurst index of the acoustic signals. Ahmad Sajjad [61] and others put forward a technology for pipeline leakage detection using acoustic emission signals. In order to identify the leakage status of pipelines, the feature vectors were input into a shallow artificial neural network. The proposed method was validated by utilizing a dataset obtained from an industrial pipeline testing platform. The algorithm presented exhibits high classification accuracy in detecting leaks of different sizes and fluid pressures. Ullah Niamat [62] and others put forward a platform based on machine learning, which is used to detect leaks with various pinhole sizes by using the channel information of acoustic emission sensors. Statistical measures are extracted from acoustic emission signals as the characteristics of the training machine learning model, and finally, the overall classification accuracy is 99%, which provides reliable and effective results suitable for implementing the proposed platform. Peng Zhang et al. [63] put forward a method of leak detection using ground acoustic signals and developed a new type of intelligent leak detection equipment according to this principle. Zhang Kang [64] and others designed an experimental system to study the characteristics of leaked sound signals. The formation mechanism of the leakage sound source is introduced, and the corresponding theoretical research is reviewed below. The effects of pipeline pressure, leakage aperture, and detection distance on acoustic signal characteristics were also studied. The results show that with an increase in internal pipeline pressure, the intensity of the leaked sound signal first increases and then decreases. With an increase in leakage aperture, the intensity of the leakage sound signal will also increase. In a short distance, the intensity is consistent regardless of the detection distance. The results of this experimental study can guide the acoustic internal detection of pipelines. This study is of practical significance for finding tiny leaks in pipelines in time and preventing leakage accidents.

#### 3.2.2. Humidity Sensor

In pipeline rupture detection, humidity sensors are usually installed in key positions near the pipeline or in areas where leakage may occur. When the sensor detects that the moisture content exceeds the set threshold, it will trigger an alarm system or send a signal to the relevant monitoring equipment. In a word, the humidity sensor plays a vital role in pipeline rupture detection, which can monitor the change in environmental humidity and moisture content in real time and give an alarm in time to ensure the safe operation of the pipeline system.

Al Fuhaid et al. [65] developed a method based on humidity sensors to detect water leakage in underground water supply pipelines. Choi Jungyu et al. [66] proposed a convolutional neural network (CNN) model for detecting and classifying leaks using vibration data collected by leak detection sensors installed in water pipes. Sadeghioon et al. [67] used humidity and temperature sensors to detect leakage. Several days of data were collected from humidity sensors and temperature sensors. By analyzing the pressure distribution map and temperature data, they concluded that there was a leak. Ahmad Abusukhon [68] and others proposed an efficient Internet of Things system for water pipeline leakage detection based on shielded pipelines, MCU nodes, and soil moisture sensors. They also created a detection system and then used various types of soil to test and evaluate the proposed system. This paper also compares several strategies for pipeline leakage detection, including the proposed system. The results show that, compared to the previous work, the time required for detecting water pipe leakage is reduced by 70%, and the hardware cost of the system is reduced by 83%.

#### 3.2.3. Ground-Penetrating Radar

Second, we can also use underground radar and geophysical prospecting technology to detect leakage in buried pipelines. Underground radar can detect leakages non-destructively by sending electromagnetic waves and identify abnormal humidity and density in the soil around the pipeline. Geophysical prospecting technology determines whether there are leaks in the pipeline by measuring the physical characteristics of underground structures, such as resistance and conductivity. Cho Younki [69] and others used standardized sampling methods to collect site characteristics such as surface coverage and spatial size, soil samples, and gas concentration data from more than 70 gas leakage sites and analyzed the collected soil samples to measure soil texture, permeability, and humidity. Statistical analysis was carried out to evaluate the influence of soil characteristics on methane migration distance and concentration. Xu Maoxuan et al. [70] designed a three-channel ground-penetrating radar (GPR) detection device for drainage pipelines, which realized the synchronous detection of the interior, main body, and external environments of pipelines and improved the detection depth and efficiency. Savin Adriana et al. [71] used ground-penetrating radar to detect and evaluate underground drainage pipes passing through large building areas parallel to the riverbed. Ayala-Cabrera, David, and others [72] used ground-penetrating radar to solve the problem of pipeline leakage in the water distribution system. Mohamed Gamal et al. [73] used a 600-MHz antenna to conduct a ground-penetrating radar (GPR) field investigation.

The simulated image generated by numerical modeling was compared to the radar profile obtained by using ground-penetrating radar in a specific position. Numerical simulation and the radar profile show that water leakage from different pipelines will have obvious effects, and the existence of saturated soil will lead to the interruption of the reflection of saturated soil waves. In the new application, envelope and migration technologies are adopted to accurately distinguish different pipeline types, especially for leakage areas. In the simulation model, there is a strong correlation between the real radar profile and the specific signal of water pipe leakage, which shows that GPR is a reliable non-destructive geophysical method for detecting water pipe leakage and distinguishing different pipeline materials under various field conditions.

## 4. Subgrade Slope

### 4.1. Distress

Subgrade slope distress refers to problems such as sliding, collapse, erosion, cracks, and subsidence in highway or railway traffic construction. These distresses may be caused by excessive slope, poor soil quality, water erosion, or changes in groundwater level, which will damage the shape and stability of slopes and pose potential risks to traffic safety and the surrounding environment. The instruments for the disease-related detection of the subgrade slope and the reference article numbers are shown in Table 3.

Subgrade slope collapse refers to the situation in which the geological structure of the subgrade slope is damaged, deformed, and slipped at the road slope due to human or natural factors, which leads to the instability or decline of the subgrade slope [74]. Poor design and construction quality of subgrade slope, improper control of parameters such as verticality, levelness, slope and standard thickness, large earthwork excavation, and poor supporting measures, or natural disasters such as rainfall, flood, landslide, and earthquake will also have an important impact on subgrade slope, which will lead to the problem of subgrade slope collapse.

**Table 3:** Representative equipment for subgrade slope.

Structure Type	Distress	Sensor	References
Subgrade slope	Subgrade slope	Global Navigation Satellite System (GNSS)	[75,76,77,78,79]
		Buried sensors	[80,81]
		Terrestrial Laser Scanning (TLS)	[82,83,84,85]
	Slope crack	Buried sensors	[86,87,88,89,90]

Subgrade slope crack refers to the crack phenomenon that appears on the road or railway subgrade slope. These cracks are usually caused by geological conditions, natural environments, or human activities. The formation of cracks may be due to a change in geological structure, soil erosion, hydraulic action caused by rainfall, earthquakes, and other reasons. The appearance of cracks may lead to the instability of subgrade slopes and then affect the safety and stability of roads or railways.

### 4.2. Monitoring Technology

Table 3 summarizes the representative research findings related to subgrade slope distress detection, and the detailed applications are elaborated in the following subsections.

#### 4.2.1. Global Navigation Satellite System

GNSS (Global Navigation Satellite System) is a technology that uses satellite signals for positioning and navigation. In the detection of subgrade slopes, GNSS can be used to monitor the deformation and stability of subgrade slopes in real time, thus helping to prevent and reduce slope disasters. GNSS technology can also be used for the long-term monitoring and analysis of slopes. Through long-term data accumulation and analysis, we can better understand the deformation law and trend of slope and provide more accurate references for slope maintenance.

He Liming et al. [75] used a GNSS to collect landslides related to steep slopes of subgrade slopes, and the experiments showed that GNSS data have wide applicability. Song Chung R et al. [76] used GNSS technology to monitor the slope with high plastic soil, and this method can provide an extremely high-resolution deformation profile. Tiwari et al. [77] made three field visits to the site by using a GNSS, collected geodetic data, and built three digital elevation models based on this to estimate the movement of the landslide’s steep slope. Ma Jun et al. [78] set up GNSS monitoring points on both sides of the subgrade slope and judged subgrade landslide distress according to GNSS data. Chen Zi et al. [79] considered that the degradation of landslide strength is directly reflected in surface displacement. Based on the GNSS shallow real-time displacement monitoring sequence, a landslide early warning method based on the GNSS displacement rate combined with the GNSS displacement tangent angle model is proposed, and early warning thresholds of different warning levels are designed. The actual monitoring layout diagram is shown in the following figure.

**Figure 7:**
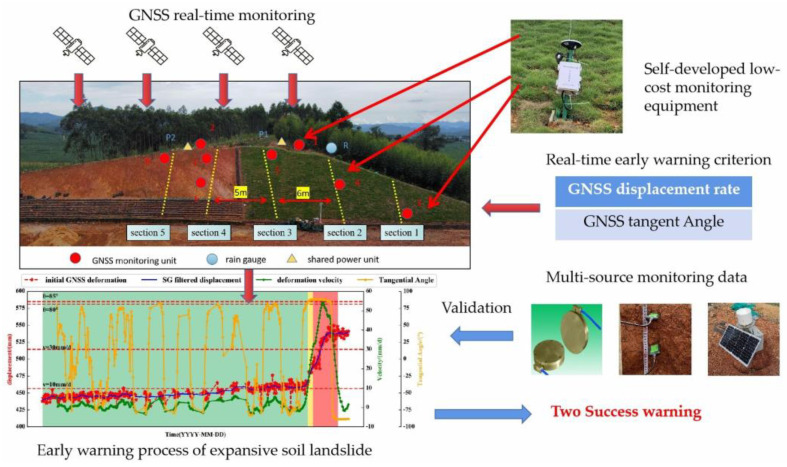
Performance monitoring of roadbed slope based on GNSSs (figures are reproduced from Chen et al. [79]).

Combined with multi-source data such as soil moisture, soil pressure, and rainfall, the feasibility of accurate landslide early warning based on GNSS real-time surface displacement is verified. The method provided by the invention does not need to carry out numerical calculations on internal stress. The landslide early warning was successfully realized twice in the test area, and the experiment proved that the method has a certain popularization value. The monitoring chart and data chart of GNSS are shown in Figure 7.

#### 4.2.2. Buried Sensors

For the phenomenon of subgrade slope collapse, researchers also use sensors such as displacement meters, earth pressure boxes, and pore water pressure gauges. The comprehensive use of displacement meter, earth pressure box, and pore water pressure monitoring technology can provide a better understanding of the deformation, soil pressure, and hydrological conditions of the slope and provide a scientific basis for monitoring, early warning, and risk assessment of slope slip.

**Figure 8:**
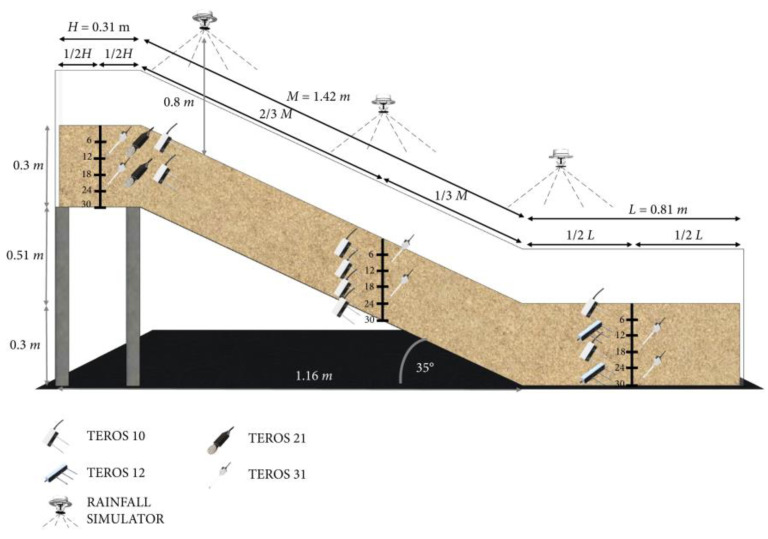
Slope sensor burying diagram (figures are reproduced from Martina et al. [81]).

Zhong Wei et al. [80] set up a physical experimental model of subgrade slope based on similarity theory and direct shear test data and observed the deformation, earth pressure, and pore water pressure of subgrade slope with sensors such as displacement meter, earth pressure box, and pore water pressure gauge. By providing surface runoff and bottom-pressure water to the slope to simulate intermittent rainfall, the sliding speed of the whole slope model is accelerated. The results show that the failure of the slope began with the toe of the slope. Then, a sliding fault occurred in stages, which extended the unstable area to the rear edge of the slope. With the continuous infiltration of rainwater, the pore pressure increased, and the matrix suction and effective stress on the bedrock surface decreased, resulting in slope failure. Martina Vivoda Prodan et al. [81] conducted a small-scale slope modeling to evaluate the failure process of landslides caused by artificial rainfall. The model platform is 2.3 m long, 1.0 m wide, and 0.5 m deep, which is used to construct small-scale slope models with the same geometric conditions but different soil types/materials, including sand and two kinds of sand and kaolin mixtures with the same slope angle. Using volume water content, pore water pressure, and matrix suction sensors installed at different depths and profiles, the hydraulic response of the slope model under simulated rainfall conditions is monitored, and the scene experimental diagram is shown in the following figure. The sensor layout diagram of the slope experiment is shown in Figure 8.

Based on the data monitoring of slope surface deformation and failure development, the factors affecting the initiation and spread of landslide and their relationship with slope materials, infiltration process, and overall soil resistance are discussed. These factors are related to soil strength, effective stress, and matrix suction contribution of the unsaturated part of the slope. Rainfall infiltration leads to an increase in volume water content, and the initial suction of partially saturated materials in the small-scale slope model disappears, which leads to a decrease in effective stress and shear strength, which in turn leads to slope movement and failure. According to the test results, the main observation results are related to the beginning and development of the observed instability of sandy and clayey slopes. The test results show that in the slope built due to the failure of clean sand, the groundwater level at the foot of the slope rises and goes back to the top of the slope, while in the slope built by sand-kaolin mixture, the instability appears in the form of cracks, which is the result of the dissipation of matrix suction caused by rainfall infiltration.

For slope cracks, the researchers detected slope cracks by embedding multiple sensors into the ground and distributing them evenly along the depth direction. The sensors can record data such as temperature, strain, pressure, and displacement at different depths of the ground and transmit these data to the data collector. The data collector can process and analyze these data, thus inferring the location and size of cracks. When cracks occur, because the soil under the surface is stretched or squeezed, the sensor will record the corresponding strain and displacement data, thus providing information about the cracks.

**Figure 9:**
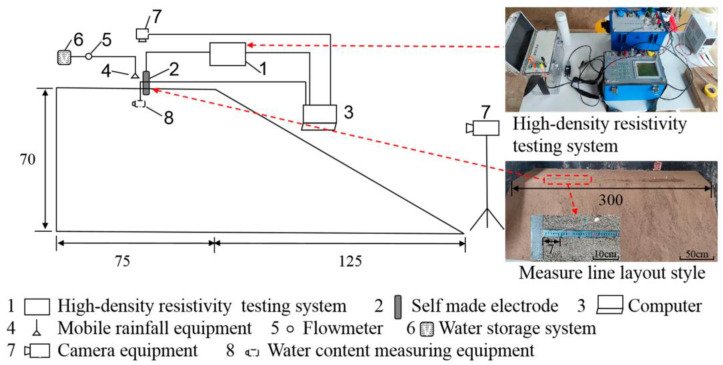
Slope test model design drawing (figures are reproduced from Kang et al. [85]).

I W Arya et al. [82] simulated the influence of water saturation on the development of cracks on the slope with cracks. Hou Hengjun et al. [83] deduced the stress mechanism of the initiation and development of main cracks by constructing a large-scale clay slope model of arc sliding surfaces. In the model test, the soil pressure sensor and displacement meter are used to monitor the development of internal stress and the horizontal displacement of the slope shoulder, respectively, under the set load sequence. Chen Xuanyi et al. [84] studied the crack propagation law of expansive soil slopes under dry and wet conditions and the influence of cracks on the slope through large-scale indoor slope tests of dry and wet cycles. The moisture content sensor was used to monitor the changes in soil moisture content at different depths during the dry–wet cycle, and the crack depth detector was used to measure the changes in crack depth in expansive soil during drying. Kang Shen Sheng et al. [85] designed the physical model of the slope to study the evolution of the source of geological disasters on the slope. The apparent resistivity of rock mass and soil on the intermittent slope is measured by the high-density resistivity method, and the formation process of disaster sources inside the slope is obtained. The design diagram of the slope experimental model is shown in Figure 9.

Based on the sensor data, the change in the characteristics of the resistivity of slopes with weak surfaces and cracks are analyzed. The evolution process of slope geological disaster sources is summarized. The resistivity response mechanism equation of the slope disaster source is derived, and the rationality of the model is verified. The test results show that the behavior of the slope conforms to the saturated-unsaturated dynamic cycle process. Apparent resistivity is positively correlated with the formation of pores and cracks on the slope and negatively correlated with the water content in the slope. In the process of fracture development, the apparent resistivity increases, while it decreases during water seepage. In the process of slope failure and disaster, the apparent resistivity will be different under the coupling effect of crack formation and seepage. In the process of geological disaster source formation, the apparent resistivity will suddenly change and fluctuate. Therefore, according to the sudden change and abnormal fluctuation of apparent resistivity detected, the development of slope geological disaster sources can be determined.

#### 4.2.3. Terrestrial Laser Scanning

TLS (terrestrial laser scanning) is a method of three-dimensional measurement by laser scanning technology. When monitoring the landslide distress of subgrade slope, TLS can be used to obtain the three-dimensional point cloud data of subgrade slope surface, analyze and process them, and obtain the information of slope shape, deformation, and displacement to realize the monitoring and early warning of landslide distress.

**Figure 10:**
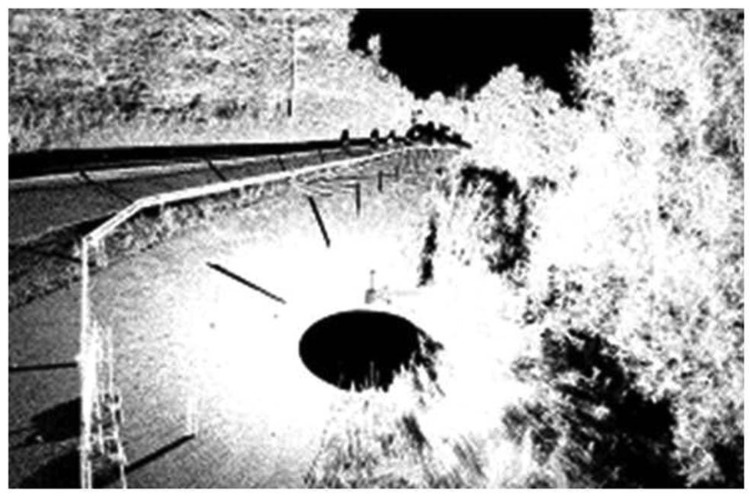
The surface of the road embankment represented by the cloud of points (figures are reproduced from Kogut et al. [90]).

Luo Lihui et al. [86] used TLS to scan the point cloud data of the slope and identify and warn of the deformation of the slope structure in real time. Abdelhalim et al. [87] pointed out that the method of evaluating the strength of a weak slope by using a ground laser scanner makes the evaluation faster, safer, and more systematic. Kopras Marek et al. [88] used a laser scanner to obtain the complete data of subgrade slope geometry and analyzed the slope stability according to the data. Zheng Xiangtian et al. [89] used a ground laser scanner to output the landslide as point cloud data, built a three-dimensional model of the main hidden arc of secondary sliding on site, and provided data for the residual dangerous rock mass according to the surface deformation monitoring, which provided support and a decision-making basis for the slope instability research. Kogut et al. [90] used ground laser scanning to detect unsafe behaviors of slopes and steep slopes. It is also convenient to evaluate the stability of earthwork. The three-dimensional point cloud scene obtained by TLS collection is shown in Figure 10.

They also pointed out that TLS can remotely detect surface changes in a simple and automated way. The laser scanner is used for routine multiple measurements to monitor the behavior of selected objects for a long time. The data are substituted into the discrete numerical model of the finite element method, the geotechnical characteristics of the matrix are considered, and the risk assessment and stability test of this kind of structure are carried out. The numerical model of the structure and substrate parameters are introduced to analyze stress, strain, displacement, and different loads.

## 5. Existing Protective and Retaining Structure

### 5.1. Distress

The existing protective and retaining structures mainly include retaining walls, anti-slide piles, and prestressed anchor cables, and the possible distresses of retaining walls include wall inclination, cracks, and settlement. The possible distresses of anti-slide piles include deformation, corrosion, anchorage failure, and so on. The possible distresses of the prestressed anchor cable include cable body fracture, anchor point failure, and corrosion. The instruments for the disease-related detection of the existing protective and retaining structure and the reference article numbers are shown in Table 4.

A retaining wall is a kind of structural engineering used to support soil, and cracks may occur in the retaining wall during use. This kind of distress is due to the uneven internal stress of the retaining wall caused by a horizontal load or an earthquake load, which makes the wall crack [91]. Cracks in retaining walls will not only affect the bearing capacity of retaining walls but also pose a potential threat to the surrounding environment and building safety.

**Table 4:** Representative equipment for existing protective and retaining structures.

Structure Type	Distress	Sensor	References
Existing protective and retaining structures	Retaining wall crack	Unmanned aerial vehicle (UAV)	[92,93,94,95,96,97]
	Retaining wall slippage	Light detection and ranging (LiDAR)	[98,99,100,101]
	Anti-slide pile displacement	Earth pressure box	[102,103,104,105,106,107]
		Distributed optical fiber	[108,109,110]
	Anchor point failure	Distributed optical fiber	[111,112,113,114,115]

The sliding distress of the retaining wall is a common problem in civil engineering, which refers to the instability and sliding of the retaining wall under the action of lateral force. This kind of distress usually occurs on the retaining wall structure under the action of external forces such as soft soil, large slopes, earthquakes, or rainfall.

The distress of anti-slide pile displacement refers to the unexpected movement or deformation of the anti-slide pile in the process of use. This kind of distress may threaten the stability of the slope and increase the risk of landslides. There are various distresses of anti-slide pile displacement, including pile deformation, horizontal displacement of pile top, pile inclination, and so on. The main reasons may be the damage of pile material, instability of pile foundation, and vibration load.

The failure distress of the anchorage point of a subgrade retaining structure refers to the phenomenon that the anchorage point in a subgrade retaining structure is damaged or failed. Anchorage points are usually used to fix subgrade retaining structures to resist the thrust and lateral force of soil. However, due to various factors, such as material aging, improper design, construction quality, and so on, the anchorage point may be damaged or unstable, which leads to its inability to effectively fix the retaining structure.

### 5.2. Monitoring Technology

Table 4 summarizes the representative research findings related to existing protective and retaining structure distress detection, and the detailed applications are elaborated in the following subsections.

#### 5.2.1. UAV

Unmanned aerial vehicles (UAVs) can quickly and accurately acquire images or point cloud data of slope surfaces by carrying a high-resolution camera or LiDAR [116]. Using the image data obtained by aerial photography of unmanned aerial vehicles, combined with image processing and computer vision algorithm, the slope cracks can be comprehensively and efficiently detected and analyzed. By identifying and measuring the parameters such as the length, width, and direction of cracks, the slope cracks can be found early.

Chern Sheng et al. [92] put forward an innovative integrated learning method in the dynamic imaging system of unmanned aerial vehicles. The retaining wall was patrolled in the flight path setting of an unmanned aerial vehicle, and then the horizontal image was immediately returned by using the wireless transmission of the system. They also classified the data to identify the cracks in the retaining wall. The method of combining machine vision with UAVs, proposed by Beskopylny et al. [93], has high universality in detecting and monitoring retaining walls. Kim et al. [94] used unmanned aerial vehicles to photograph various defects such as cracks, holes, weathering, wet patches, and peeling in retaining walls and used an improved target recognition algorithm to classify the types of defects. Kaartinen et al. [95] mentioned that LiDAR has great damage detection potential and introduced the latest progress in structural health monitoring based on LiDAR, especially for detecting cracks, deformation, defects, or structures. Deligiannakis et al. [96] measured the retaining wall structure by an unmanned aerial vehicle equipped with laser radar and inferred the location and severity of cracks according to the point cloud data. Dong Han et al. [97] combined machine vision with unmanned aerial vehicles (UAVs) to detect cracks in the retaining walls of mountain climbing areas or forest roads. Using the image of the retaining wall collected by UAVs in advance, the gap of the retaining wall is obtained as the target of sample data. After repeated training of the deep learning neural network module, the characteristic conditions of cracks are extracted from the image to be measured. Then, various features of gap features are extracted by image conversion, and the influencing factors are analyzed to evaluate the risk degree of the gap.

A series of gap risk factor equations are proposed to analyze the security of the detected gap images so that the system can judge the image information collected by the UAV and assist users in evaluating the security of the gap. At present, deep learning modules and gap hazard assessment methods are used to make suggestions for gaps. The expansion of the database effectively improves the efficiency of gap identification. The detection process is about 20~25 frames per second, and the processing time is about 0.04 s. In the process of capture, there will still be some misjudgments and incorrect circle selection. The misjudgment rate is between 2.1% and 2.6%.

#### 5.2.2. LiDAR

Laser radar can quickly and accurately obtain the point cloud data on the surface of the retaining wall. By using the point cloud data obtained by laser radar, combined with point cloud processing and computer vision algorithm, the slope distress of the retaining wall can be comprehensively and efficiently detected and analyzed. By identifying and measuring the parameters such as the inclination degree and direction of the retaining wall, the inclination problem of the retaining wall can be found early, and targeted repair measures can be provided. Compared to traditional measurement methods, LiDAR has the advantages of flexible operation, wide coverage, high speed, and high safety, which can provide more accurate and comprehensive data support for the management and maintenance of retaining walls and improve work efficiency and safety.

**Figure 11:**
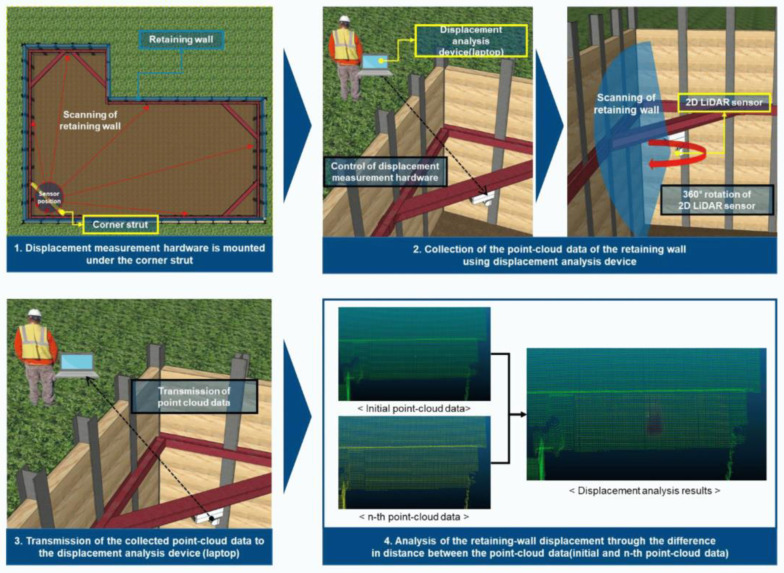
Measurement method of the 2D LiDAR sensor-based retaining wall displacement measurement system (figures are reproduced from Jun et al. [101]).

Liu Zuguang et al. [98] reconstructed the retaining wall in 3D based on LiDAR, processed it based on the collected data, and obtained the overall running speed and accuracy. Malka et al. [99] built comprehensive 3D data of retaining walls based on LiDAR and considered the influence of human factors on distresses. Vishal et al. [100] mentioned the use of LiDAR to detect slopes to prevent landslides. Jun Sang et al. [101] developed a retaining wall displacement measurement system based on a two-dimensional (2D) LiDAR sensor, which is convenient for installation and three-dimensional displacement detection. The developed system collects 360-degree point cloud data about the retaining wall by rotating the 2D LiDAR sensor at a constant speed. The detection system of retaining wall tilt based on 2D scenario is shown in Figure 11.

To evaluate the displacement measurement performance of the system, laboratory experiments were carried out using the simulated deformation model. The root mean square error of the system is 2.82 mm, and the results show that the system is economically feasible.

#### 5.2.3. Earth Pressure Box

Earth pressure box is a commonly used instrument, which senses the lateral pressure of soil against sliding piles. When the anti-slide pile is displaced, the pressure exerted by the soil will also change. Then, through the data acquisition system connected to the earth pressure box, the pressure signal in the earth pressure box is recorded and monitored in real time. These signals can be converted into corresponding displacement data, and the displacement of anti-slide piles can be determined through calculation and analysis.

**Figure 12:**
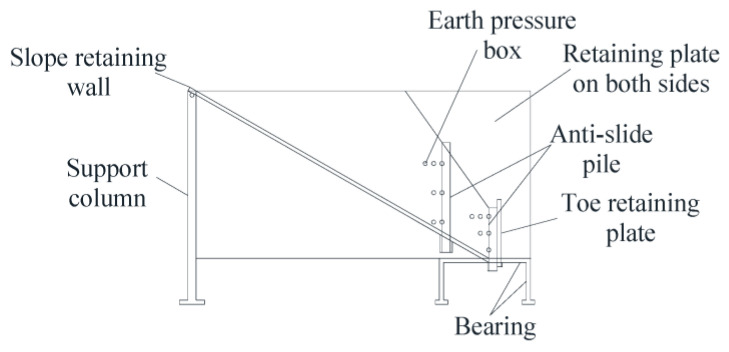
The model’s assembled profile (figures are reproduced from Zhang et al. [107]).

Jin Honghua et al. [102] built a test system for the bearing characteristics of cantilever anti-slide piles and carried out a physical model test for the bearing characteristics of cantilever anti-slide piles under a trapezoidal thrust load. In the course of the experiment, the earth pressure data collected by the earth pressure box were used as the standard to judge its displacement. Liu Xunchang et al. [103] put forward the internal force solution model of the anti-slide short pile based on the data from the earth pressure box and finite element method and tested the model in the laboratory. By comparing the internal force and deformation of these piles, the test verifies the proposed calculation model of anti-sliding short piles. Ren Xiang et al. [104] studied the relationship between the stress of an anti-slide pile, the bending strain of the pile, and the soil stress in front of the pile and the load. Based on the data of the strain gauge and earth pressure box in the model test, the three-dimensional morphological changes in the passive earth arch in front of the pile are studied by the numerical simulation method. Zhong Wei et al. [105] carried out a series of model pile pushing and direct shear tests, using earth pressure to detect the change in earth pressure during the test, and the test results were used to design the relative displacement monitoring system. Wang Hao et al. [106] developed a method to analyze anti-slide piles in two bedrocks with different strengths under the sliding surface based on the data of the earth pressure box. Zhang Sifeng et al. [107] developed an indoor model experimental device to conduct a detailed study of the action mechanism of a prestressed anchor anti-slide pile and established a finite difference and particle flow numerical analysis model of a slope anchor anti-slide pile based on a road slope reinforcement project. The buried diagram of the earth pressure box in the retaining wall is shown in Figure 12.

Based on the analysis of the stress and displacement characteristics of the anti-slide pile using the data of the earth pressure box, the influence of the prestress of the anchor cable, the inclination angle of the anchor cable, the width and column spacing of the anti-slide pile, the inclination angle of the landslide, height, and the nature of the fill on the stress and deformation characteristics of the pile is discussed, and some design parameters are optimized. The results show that the greater the prestress of the anchor cable, the smaller the displacement of the pile, but the excessive stress is not conducive to the safety of the pile. The optimum tension should be 50–70% of the design tension of the anchor cable. With the increase in the inclination angle of the anchor cable, the displacement of the pile first decreases and then increases, and the optimal inclination angle of the anchor cable is obtained. In double-row piles, with the increase in pile spacing, the front row piles gradually change from supporting the soil between the double-row piles to supporting the sliding body with the back row piles, and the double-row piles are plum blossom-shaped. When the pile spacing is 2.5 times the pile diameter, the force of the front and rear piles is the most reasonable.

#### 5.2.4. Distributed Optical Fiber

The distributed optical fiber sensor is a new detection technology based on the optical principle, which can be used to monitor the displacement and strain of various structures. In the displacement detection of the anti-slide pile, distributed optical fiber sensors are arranged along the anti-slide pile device, and the continuous distribution curve of anti-slide pile displacement can be obtained in real time by injecting a pulsed laser beam and processing the reflected light signal.

**Figure 13:**
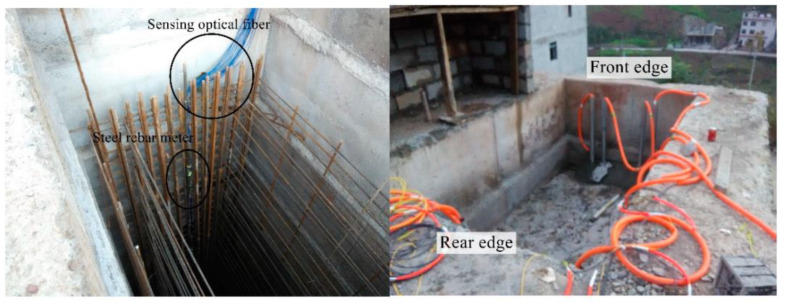
Site construction of the anti-slide pile (figures are reproduced from Wei et al. [110]).

Zhang Meng et al. [108] monitored the soil pressure near the anti-slide pile by fixing the pressure fiber grating sensor on the anti-slide pile. Cheng Gang et al. [109] fully considered the soil stress conditions of anti-slide piles and selected distributed optical fiber as a monitoring instrument according to its characteristics. Wei Chaoqun et al. [110] pointed out that the long-term monitoring of anti-slide piles can help to understand the process of anti-slide piles subjected to landslide thrust; so, distributed optical fiber sensing technology was used to study the internal force of anti-slide piles under long-term landslide thrust. Figure 13 shows the installation diagram of distributed optical fiber on site.

After obtaining the distributed optical fiber data, the BP neural network is used to train the model according to the monitored strain value and the calculated bending moment value. The results show that the monitoring results of the sensor optical fiber reflect the actual situation more accurately than the steel bar meter and can locate the sliding area more accurately. Under the long-term action of landslide thrust, the bending moment distributed on anti-slide piles occurs in stages. According to the development trend of bending moment value, three stages can be summarized. These three stages can be divided into two change periods of landslide thrust; the model generated by BP neural network training can predict the bending moment. In this paper, the long-time interval sensing optical fiber monitoring provides a basis for the long-term performance analysis of anti-slide piles and the stability evaluation of landslides. Using the BP neural network to train related data can provide the direction for future engineering monitoring.

**Figure 14:**
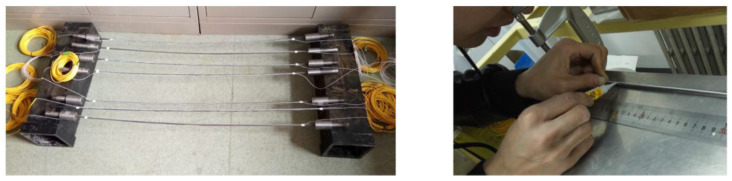
Deployment of distributed optical fibers (figures are reproduced from Li et al. [115]).

For anchor point failure, Gao Lei et al. [111] used distributed optical fiber technology to obtain the strain distribution of prestressed anchor cable and analyzed the distribution law of axial force. Guo Gaochuan et al. [112] proposed a new method to detect the quality of prestressed anchor cable based on the fiber Bragg grating sensing principle. The strain transfer mechanism between surface-bonded fiber Bragg grating and prestressed anchor cable is also studied. The optical fiber monitoring and testing platform of prestressed anchor cable quality is established. Il-Bumkon and Yong-seokkon et al. [113,114] put forward a method to monitor the anchorage force by measuring the strain field on the bearing plate with distributed optical fiber sensors and, through experiments, showed that the anchorage force has a great correlation with the strain distribution on the bearing plate. Li Jianzhi et al. [115] developed an axial distribution test method based on the high-stress characteristics of prestressed anchor cables and used distributed optical fibers to test the corrosion damage to prestressed anchor cables. Figure 14 shows the distributed optical fiber layout.

Based on distributed optical fiber data, the positioning accuracy and corrosion range of axial distributed optical fiber sensors are studied, and the mathematical model between corrosion quality loss and axial fiber strain is established. The experimental results show that the fiber strain from the axial distribution sensor enables people to reflect the corrosion rate on the prestressed anchor. In addition, when the anchored cable has higher stress, it has higher sensitivity.

## 6. Discussion

The detection of subgrade distresses holds significant importance for fostering sustainable development and resilient transportation systems. This review centers its attention on sensor devices utilized for subgrade distress detection and provides comprehensive insights into representative detection technologies from diverse perspectives. This review encapsulates the fundamental principles and applications of sensors employed in detecting distresses across various road structures, accompanied by a detailed examination of their technical attributes. In light of existing problems and the development needs of intelligent transportation, this discussion categorizes detection methodologies into remote sensing detection, ground-based detection, and embedded sensor detection. It considers these directions as deserving of further research attention.

### 6.1. Remote Sensing Detection

The remote sensing detection technologies mentioned in this review include InSAR, a GNSS, and unmanned aerial vehicles. Typically, the detection of long-distance distresses is associated with the overall deformation and displacement of road structures. For instance, InSAR technology is adept at detecting subgrade subsidence distresses, while a GNSS is particularly effective for detecting subgrade slippage. Similarly, unmanned aerial vehicles are employed for the detection and identification of cracks in retaining walls. Leveraging these remote sensing detection methodologies enables the large-scale, real-time monitoring of road health. Nevertheless, current remote sensing detection technologies exhibit limitations in accuracy. Consequently, future development in this field will focus on enhancing various aspects, including increasing the temporal and spatial resolution of data, as well as refining resolution and accuracy.

### 6.2. Ground Detection

The instruments employed for detecting subgrade distresses on the pavement, as discussed in this review, encompass ground-penetrating radar, total station, falling weight deflectometers, terrestrial laser scanning, and LiDAR. This detection method can use non-destructive testing to detect the deformation and strength within the subgrade, such as with ground-penetrating radar, total station, and falling weight deflectometer equipment. Additionally, it entails the external scanning of supporting structures and subgrade slopes to enable further analysis of distress types. While this pavement-based method of subgrade distress detection exhibits higher accuracy compared to remote sensing detection technologies, its capability is limited to detecting distress types within a specific distance or at a particular point. As a result, it falls short of achieving a comprehensive detection of distresses along the entire road. Therefore, future advancements should not only focus on enhancing the accuracy and intelligence of the detection process but also on expanding the detection range of instruments to enable broader applications on a larger scale of roads.

### 6.3. Embedded Sensor Detection

Embedded sensors primarily encompass distributed optical fibers, sound sensors, humidity sensors, and earth pressure boxes, among others. These sensors exhibit higher detection accuracy as they are buried beneath the pavement and are directly in contact with the subgrade. The embedded sensors listed in this paper, apart from distributed optical fibers, are limited in their ability to detect distresses only within a specific distance or at certain points. Additionally, the deployment of embedded sensors necessitates precise burial at specified depths within the subgrade during the construction phase. Consequently, the establishment of detailed embedding regulations becomes imperative to ensure the detection accuracy and range of the embedded sensors in practical engineering applications. Distributed optical fibers offer notable advantages such as high detection accuracy and a wide detection range. However, their capability is primarily limited to detecting subgrade settlement data. Therefore, there exists substantial research potential in exploring long-distance and high-precision detection techniques for other parameters of the subgrade, including water content and stress parameters.

When using these sensors for road subgrade distress detection, the implementation cost and economic return need to be considered. The implementation cost includes expenses for equipment purchase, installation, personnel training, and data processing. High-precision sensors such as ground-penetrating radar, InSAR, LiDAR, and terrestrial laser scanning have high equipment costs, require specialized training for operators, and need professional technical support for data processing, resulting in high implementation costs. On the other hand, common sensors like sound sensors, humidity sensors, and soil pressure boxes have relatively lower equipment costs, but the cost of equipment quantity and deployment still needs to be considered for large-scale applications. For example, in the case of distributed fiber optic sensors, the cost of laying the fiber optic cable is low in the overall project, but the cost of the fiber optic demodulator is high. Therefore, when facing extensive road subgrade distress monitoring, distributed fiber optics would be a better choice. For post-construction road distress detection, ground-penetrating radar, InSAR, LiDAR, terrestrial laser scanning, and falling weight deflectometers (FWDs) are more suitable. For roads under construction, with specific locations requiring focused parameter detection, buried sensors such as sound sensors, humidity sensors, and soil pressure boxes are more suitable.

Presently, the three-dimensional monitoring of subgrade facilities implies a large amount of cross-modal data, which may be presented in diverse formats and lack standardized encoding rules. While the partial implementation of three-dimensional monitoring has been achieved in existing research, the prevalent issue of data isolation persists, resulting in low utilization rates of the data. In this scenario, the complete potential of the three-dimensional monitoring system remains underutilized. Consequently, there exists an urgent imperative to investigate and deploy unified encoding mechanisms and swift fusion techniques for integrating multi-source heterogeneous monitoring data. From a decision-making standpoint, three-dimensional monitoring facilitates the provision of multi-source information. However, prevailing decision-making subsystems predominantly operate independently. In essence, closed data impede effective data sharing, complicating the update and collaboration among monitoring subsystems, thereby undermining the optimal decision-making of the entire system. Certain monitoring tasks exhibit interrelatedness in terms of feature morphology and occurrence mechanisms. Therefore, the adoption of a multi-learning framework presents a viable approach to fully explore complex information from related tasks, thereby optimizing decision-making strategies.

## 7. Conclusions

This paper systematically summarizes and presents diverse methodologies for the detection of subgrade distresses, meticulously analyzing their respective advantages and limitations. Through this comprehensive examination, this paper furnishes comprehensive guidance. The research results presented in this paper underscore the pivotal role of the timely and accurate detection of subgrade distresses in improving the longevity of roads, mitigating financial burdens, and safeguarding traffic safety. The research results of this paper provide comprehensive guidance for the detection of subgrade distresses, thereby facilitating the timely identification and tailored remediation of subgrade distresses.

In this paper, the subgrade structure is divided into four sections. A review of the research on the detection methods of potential distresses, which could occur in these four sections, was conducted. Among all the research methods, this paper references at least 14 types of sensors used in road distress detection. By summarizing and analyzing existing research, it was found that the current research direction focuses on combining non-destructive detection equipment data with artificial intelligence computing methods. While scholars have already integrated road distress detection data with artificial intelligence algorithms in method embankment and subgrade and drainage facilities, research on the integration of detection devices and artificial intelligence algorithms for subgrade slope and existing protective and retaining structures is relatively limited. Therefore, the intelligent detection of roadbed distresses in the future holds significant value in promoting the healthy service of roads and road traffic safety.

Through the adoption of advanced detection technologies and methods, improvements in the service life of roads can be realized, accompanied by reductions in financial costs, ultimately elevating the overall standards of construction and operational practices within the transportation infrastructure. Hence, subgrade distress detection plays a paramount role in ensuring traffic safety and enhancing economic benefits. The accurate detection and effective prevention of subgrade distresses are crucial for fostering road traffic safety and facilitating economic development. In summary, with ongoing technological advancements and methodological innovations, the prospect of subgrade distress detection is poised for substantial growth, promising greater contributions to the sustainable operation of transportation infrastructure and broader social development.

## Figures and Tables

**Table 2:** Representative equipment for drainage facilities.

Structure Type	Distress	Sensor	References
Drainage facilities	Pipeline leakage distress	Sound sensor	[59,60,61,62,63,64]
		Humidity sensor	[65,66,67,68]
		Ground-Penetrating Radar (GPR)	[69,70,71,72,73]
